# Comparative Lateralizing Ability of Multimodality MRI in Temporal Lobe Epilepsy

**DOI:** 10.1155/2016/5923243

**Published:** 2016-11-15

**Authors:** Karabekir Ercan, Hediye Pinar Gunbey, Erhan Bilir, Elcin Zan, Halil Arslan

**Affiliations:** ^1^Radiology Department, Ataturk Training Research Hospital, Ankara, Turkey; ^2^Radiology Department, Ondokuz Mayıs University, Samsun, Turkey; ^3^Neurology Department, Gazi University, Ankara, Turkey; ^4^Radiology Department, Johns Hopkins Medical Institution, Baltimore, MD, USA

## Abstract

*Purpose*. The objective is to compare lateralizing ability of three quantitative MR (qMRI) modalities to depict changes of hippocampal architecture with clinical entities in temporal lobe epilepsy.* Methods*. We evaluated 14 patients with clinical and EEG proven diagnosis of unilateral TLE and 15 healthy volunteers. T1-weighted 3D dataset for volumetry, single-voxel ^1^H MR spectroscopy (MRS), and diffusion tensor imaging (DTI) were performed for bilateral hippocampi of all subjects.* Results*. Individual volumetric measurements provided accurate lateralization in 85% of the patients, spectroscopy in 57%, and DTI in 57%. Higher lateralization ratios were acquired combining volumetry-spectroscopy (85%), spectroscopy-DTI (85%), and volumetry-DTI (100%). Significantly decreased NAA/(Cho+Cr) ratios (*p* = 0.002) and increased FA (*p* = 0.001) values were obtained in ipsilateral to epileptogenic hippocampus. Duration of epilepsy and FA values showed a significant negative correlation (*p* = 0.016, *r* = −0.847). The history of febrile convulsion associated with ipsilateral increased ADC values (*p* = 0.015, *r* = 0.851) and reduced NAA/(Cho+Cr) ratios (*p* = 0.047, *r* = −761).* Conclusion*. Volumetry, MRS, and DTI studies provide complementary information of hippocampal pathology. For lateralization of epileptogenic focus and preoperative examination, volumetry-DTI combination may be indicative of diagnostic accuracy.

## 1. Introduction

Temporal lobe epilepsy (TLE) is the most common type of intractable and partial epilepsies which is predominantly associated with history of febrile convulsions and anterior temporal lobe EEG abnormalities [1, 2]. Pathologic analysis shows hippocampal sclerosis in 65% of temporal lobectomy specimens from adults with TLE [3, 4]. The only standard treatment for seizure-free status with manifest TLE is surgical which includes amygdala-hippocampectomy [5, 6]. Recently Magnetic Resonance (MR) imaging techniques have had a very important role in precise identification of seizure focus in TLE patients who require surgical treatment. The surgical results of patients without any reliable abnormalities on MR imaging generally show less favorable outcome [6]. Therefore, it is necessary to detect and localize the seizure focus for the best surgical outcome. Atrophy of hippocampal formation on T1-weighted spin echo images and signal increase on fluid attenuated inversion recovery (FLAIR) images are the basic visual qualitative MR findings [7, 8].

Comparing the quantitative MR (qMR) studies of hippocampus with qualitative ones may provide more useful information to detect the severity and extent of hippocampal damage in an objective perspective. Furthermore several qMR techniques may give prognostic insight into surgical outcome. There have been different qMR techniques like volumetry, MR spectroscopy (MRS), T2 relaxometry, and diffusion weighted imaging (DWI) described to date [9–11]. In several studies volumetric measurements have demonstrated hippocampal atrophy with a high degree of accuracy [12–14]. ^1^H MRS allows evaluation of biochemical changes noninvasively in patients with intractable TLE. Diffusion tensor imaging (DTI) is an advanced MR technique that has been recently applied for localizing the epileptic focus in TLE patients [9–11]. DTI is used to indirectly evaluate the structural integrity of brain tissue by providing information about the magnitude (apparent diffusion coefficient) and direction (fractional anisotropy) of water diffusion. Hippocampal qMR studies compared volumetry, T2 relaxometry, DWI, and MRS [15–20] before. Nevertheless there is no published study correlating volumetry, MRS, and DTI findings in TLE patients.

The aim of this study was investigating relative diagnostic lateralizing value of volumetry, ^1^H MRS, and DTI findings and furthermore clinical data such as age, duration of epilepsy, febrile convulsion history, and response to antiepileptic drugs (AED) in a homogeneous group of TLE patients. The long term effects of febrile convulsions and duration of epilepsy may lead to damage in tissue microstructures. The evident hippocampal sclerosis with signal changes on conventional MR images suggests microtissue damage at the same time. The results or reasons of epilepsy may affect the micro- and macrostructures of the hippocampi which can be demonstrated by different neuroimaging MR modalities. Alterations of microstructure may be presented as decreased neuronal metabolite concentrations on MRS and tissue integrity on DTI. Volumetric analysis may also demonstrate tissue atrophy in both micro- and macrostructural changes. All of these qMR modalities may provide overlapping useful data about different hippocampal damage stages of epilepsy. One modality cannot describe the hippocampal pathologic spectrum sufficiently alone. The important question is whether these modalities can provide complementary or unnecessary information of hippocampal structures.

## 2. Materials and Methods

### 2.1. Patients and Controls

This prospective study was approved by the local ethics committee. Written informed consent was obtained from the patients and healthy volunteers participating in the study. Fourteen patients (six women and eight men with a mean age of 34.2 years (SD: 12, range 19 to 66 years)) with the diagnosis of TLE were recruited from our neurology department. The diagnosis of TLE based on the clinical examination was obtained by an experienced neurologist, the seizure semiology, interictal EEG monitoring, and previous hospitalization records. All of the EEGs were acquired in the neurology department and interpreted by an experienced neurologist. During MR evaluation, the authors were blinded to subject's identity, age, and epileptic site as determined by EEG recordings. Subsequently TLE diagnoses were confirmed by positive EEG findings. The patients with nontemporal epilepsies and other pathologies such as tumors or vascular lesions associated with hippocampal sclerosis were excluded from the study.

The control group included 15 healthy volunteers (9 women and 6 men, with a mean age of 34.8 years (SD: 12.1, range 21 to 52 years)) who did not have any history of neurological disorders and had no abnormal conventional MR findings.

### 2.2. MR Imaging Protocol

All patients were scanned on a 1.5 T MR scanner (Philips Achieva, Netherlands; slew rate 40 mT/m) with an 8-channel head coil and underwent a single MR session. During 45-minute scanning time all subjects were placed comfortably in the scanner and their heads were fixed with head coils. Symmetric positioning was performed for every subject carefully.

Standard qualitative imaging protocol for epilepsy patients included axial and sagittal T1-weighted (T1W) turbo-spin-echo (TSE) (TR/TE = 450/9 ms, slice thickness (thk): 5 mm) and T2W TSE (TR/TE = 5000/100 ms, thk: 5 mm) and coronal FLAIR (TR/TE = 6000/120 ms, IR: 2000 ms, thk: 2 mm) sequences with using a tilted orientation to the temporal lobes, orthogonal to axis of the hippocampal body axis. For hippocampal volumetry studies coronal planes parallel to the posterior commissue-obex (PC-OB) line were obtained. Single-voxel MRS of hippocampus was acquired in the axial plane perpendicular to PC-OB line.

### 2.3. Qualitative Visual Analysis

Coronal oblique thin section T1-weighted images obtained by reformatting a sagittal 3D T1 sequence through the entire brain were used for the assessment of hippocampal atrophy. The widening of temporal horn of lateral ventricle, moderate loss of hippocampal volume (decrease in height), and severe volume loss of hippocampus were assessed as atrophy. FLAIR images were used to assess the signal intensity increments for sclerosis. The qualitative evaluations were performed by two evaluators (Karabekir Ercan and Hediye Pinar Gunbey) who were blinded to the other evaluators' data.

### 2.4. Volumetry

**Figure 1:**
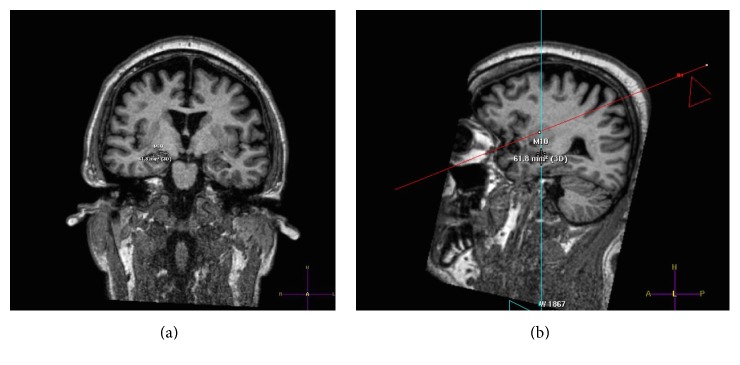
Hippocampus borders were defined from coronal planes (a) with assessing the anterior and posterior limits on the right and left parasagittal sections (b).

We performed T1W 3D gradient-echo sequence (TR = 7.2 ms, TE = 33 ms, NSA = 1, FOV = 256 mm, slice thickness 1 mm, gap = 0 mm, flip angle = 8°, and matrix = 256 × 256 pixels) through 160 slices of the brain. Hippocampus borders were defined at workstation according to procedure of Watson et al. [21]. For each subject while measuring from coronal planes, the anterior and posterior limits of each hippocampus were assessed on the right and left parasagittal sections (Figure 1). The uncal recess and alveus were accepted as the reference points for discrimination of the anterior border of the hippocampus from the amygdala. The superior border was drawn by visualization of choroid plexus and lateral border; temporal horn and medial border; and perimesencephalic cistern and posterior border, drawn according to the visualization of the crus of fornix. The inferior border was drawn when the subiculum was seen. Hippocampal volume calculations were obtained with Cavalieri method [22] which is a planimetry method by multiplying the area of hippocampus in each slice with slice thickness using Philips Achieva Extended MR Workspace R2.6.3.1. The measurements were performed by two evaluators (Karabekir Ercan and Hediye Pinar Gunbey) who were blinded to the other evaluator's data and the average of two evaluators measurements were obtained lastly for reliability.

Finally to correct the individual differences of each subject head size we used a ratio that was described by Cendes et al. [23] and modified by Kälviäinen et al. [24].

Owing to generated volumes of both right and left hippocampus, numeric criteria must be given that will divide the right and left hippocampal volume measurements into three forms: right hippocampal atrophy, indeterminate or nonlateralizing, and left hippocampal atrophy. So for right/left hippocampal volume ratio we subtracted the* left- *from* right-*sided volume of each hippocampus. This method has been used before [12] to identify asymmetry of hippocampus. Right-left difference ≤ 200 mm^3^ was counted in right atrophy; ≥ 600 mm^3^ was counted in left atrophy. Values between 200 and 600 mm^3^ were considered nonlateralizing.

### 2.5. MR Spectroscopy

The spectroscopic measurements were performed with the software of Philips Achieva Extended MR Workspace R2.6.3.1. on the workstation. In single-voxel acquisition, voxel of interest (VOI) was placed on hippocampus with sagittal, axial, and coronal orientation and was defined 20 × 20 × 20 mm for each hippocampus. Point-resolved spectroscopic (PRESS) sequence (TE = 144 ms, TR = 2000 ms, and NSA = 128) was acquired. Shimming and water suppression were performed automatically. Hippocampal metabolic signals were measured at 2.0 ppm (NAA), 3.0 ppm (Cr), and 3.2 ppm (Cho). NAA/Cr, Cho/Cr, and NAA/(Cho+Cr) ratios that were shown to be reliable in lateralization of epileptogenic focus [25] were also calculated for each hippocampus. Abnormal values were defined as 2 SDs below the mean of control subjects.

### 2.6. Diffusion Tensor Imaging

**Figure 2:**
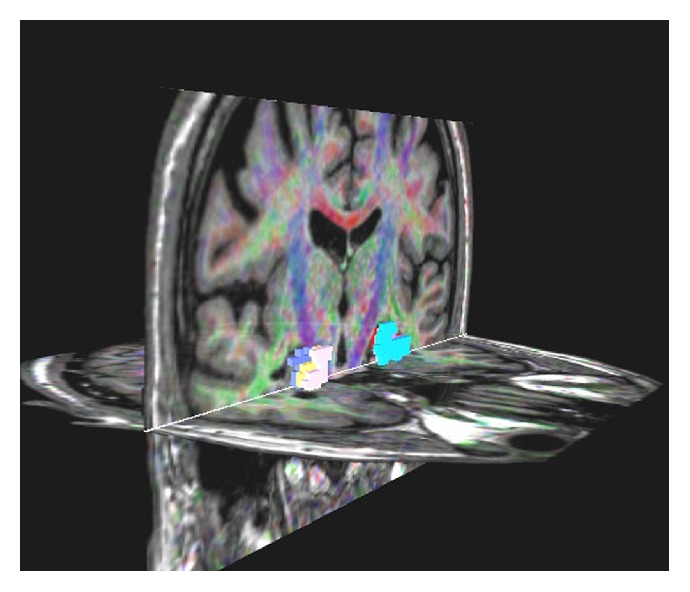
DTI study of hippocampus using ROI drawn on workstation at contiguous three slices on coronal images. FA and ADC values were obtained from the average of these three slices.

DTI was performed for all patients and control subjects. Special attention was paid to patients for being seizure-free for at least 24 hours, because seizure activity could affect diffusivity [26]. The DTI data were obtained using a single-shot spin-echo planar image (SE-EPI) sequence (matrix = 128 × 128; field of view = 256 mm with a measured voxel size of 2.69 × 2.69 × 2.7 mm and a reconstructed voxel size of 2.00 × 2.00 × 2.7 mm; TE = 90 ms, TR = 10,150.5 ms; SENSE factor = 2; EPI factor = 67; *b* = 1,000 mm^2^·s^−1^; NSA = 3; and slice thickness = 2.3 mm, gap = 0 mm). The diffusion sensitizing gradients were applied simultaneously along 32 noncollinear directions (*b* = 1000 s/mm^2^) as well as an acquisition without diffusion weighting (*b* = 0) through 60 contiguous slices parallel to the anterior commissure-posterior commissure line. Realignment of the DTI images was performed using the diffusion registration software package provided by the manufacturer (Extended MR WorkSpace, version R2.6.3.1, Philips Medical Systems). The diffusion affine registration tool (Philips) was used to remove shear and eddy current distortion and motion artifacts. The T1-weighted 3D MR images were used for anatomical reference and regions of interest (ROIs) were placed on hippocampus on workstation for three consecutive slices. Fractional anisotropy (FA) and apparent diffusion coefficient (ADC) values were measured from the average of these three slices by two evaluators blinded to the other evaluator's data (Figure 2). Lastly the average of two evaluators measurements was obtained for reliability.

### 2.7. Statistical Analysis

We used the Kolmogorov-Smirnov test to verify the normal distribution of qMRI values. For comparing the means of variables with controls we applied the equality of variances (Levene's test) and Student's* t*-test. The level of significance for all analysis was *p* < 0.05. Correlations between values were calculated separately for the ipsilateral and contralateral hippocampus with two-tailed Pearson's correlation test. To assess interreader variability, Shrout and Fleiss intraclass correlation coefficients (ICCs) were used [27] ICCs were interpreted according to the criteria of Landis and Koch [28]. The following ICC categories were used for interpretation: 0.01–0.20 indicated slight agreement; 0.21–0.40 fair one; 0.41–0.60 moderate one; 0.61–0.80 substantial one; 0.81–1.00 almost perfect one.

## 3. Results

### 3.1. Patients

Based on neurophysiological evaluation, patients were separated in two groups according to EEG lateralization: right TLE (*n* = 7) and left TLE (*n* = 7). For each subject bilateral hippocampal signal intensities, age, duration of epilepsy, interictal EEG lateralization, received AEDs, response to AEDs, and history of febrile convulsion were evaluated and summarized in Table 1. There was no difference in age (*p* = 0.53) and sex distribution (*p* = 0.46) between patients and normal subjects.

### 3.2. Qualitative Visual Analysis

On volume-acquired T1W images, 11 unilateral and 3 bilateral hippocampal atrophies were detected in 14 patients by visual analysis. There were 4 right-sided unilateral atrophies and 1 bilateral atrophy with 1 normal appearing hippocampus in right TLE group. Left TLE group showed 4 left-sided unilateral atrophies, 2 bilateral atrophies, and 1 normal appearing hippocampus.

Hippocampal sclerosis were assessed with increased signal intensity on coronal FLAIR images and found to be unilateral in seven patients (4 in right TLE group; 3 in left TLE group) and bilateral in 5 patients (3 in right TLE group; 2 in left TLE group). Two of 14 patients had bilateral normal signal intensity (in left TLE group). The ICCs for hippocampal atrophy and signal increment ranged from 0.81 to 0.93 for controls and TLE patients. According to the interpretation of Landis and Koch, this was an “almost perfect” interreader agreement for visual analyses and signal intensity [28].

### 3.3. Quantitative Analysis

Hippocampal MRS showed significant low NAA/Cr (*p* < 0.001), NAA/(Cho+Cr) (*p* = 0.002), and additionally Cho/Cr (*p* = 0.014) of right hippocampus in right TLE group compared to normal subjects. However no significant differences were found between left TLE group and controls. Nevertheless contralateral MRS abnormalities with decreased NAA (*p* = 0.031) and increased Cho (*p* = 0.043) of right hippocampus were seen in left TLE group.

In volumetric analysis after normalization of hippocampal volumetric data, 6 patients (42%) (2 in right TLE group; 4 in left TLE group) had unilateral reduced volumes and 3 patients (21%) (1 in right TLE group; 2 in left TLE group) had bilateral reduced volumes (<2 SD below mean control, *p* = 0.012), while remaining 5 patients (35%) (2 in right TLE group; 3 in left TLE group) had normal volumes.

**Figure 3:**
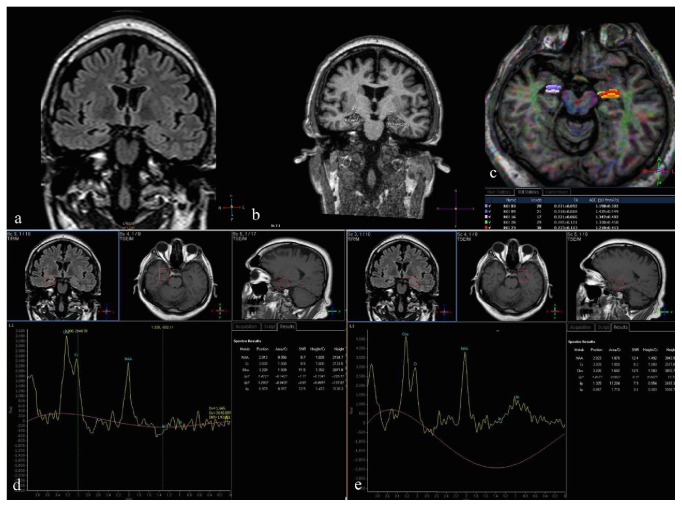
Quantitative and visual analysis of a right-sided TLE patient. Coronal FLAIR thin cut (2 mm) orthogonal to hippocampus. Right hippocampal gyrus is atrophic and shows increased intensity compared to left (a). 3D T1W coronal image shows reduced volume subjectively interpreted as atrophy. The coronal volumetric image shows reduced area on the right hippocampus as well (b). DTI and 3D T1W axial fused image shows reduced FA and increased ADC values at right hippocampus compared to left (c). Right hippocampal gyrus (d) shows reduced Cho/Cr and NAA/Cr compared to left (e) (please note that lactate is absent).

**Figure 4:**
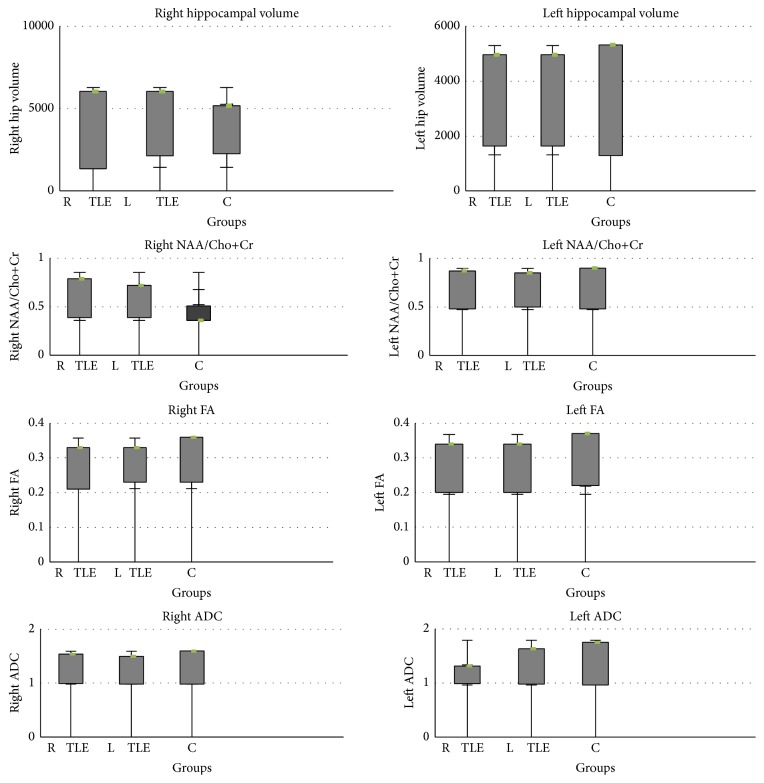
Hippocampal volumetry, spectroscopy, and DTI results.

DTI measurements of bilateral hippocampus were compared between patients and controls using statistical thresholds for FA (<2 SD below mean control, 0.23 for right hippocampus; 0.21 for left hippocampus) and ADC (>2 SD above mean control, 1.31 for right hippocampus; 1.23 for left hippocampus). Four of seven patients (57%) in right TLE group had ipsilateral reduced FA and 2 of them (28%) had ipsilateral increased ADC. In the left TLE group 2 patients (28%) showed ipsilateral reduced FA and 2 other patients (28%) showed ipsilateral increased ADC. Contralateral abnormalities were seen only in 1 patient in left TLE group representing increased ADC in right hippocampus. The results revealed significant reduced FA in right TLE group (*p* = 0.001) compared to control group. Quantitative and qualitative visual results of a right- sided TLE patient are illustrated in Figure 3. All results of analysis are summarized in Figure 4.

### 3.4. Correlations and Lateralizing Ability of MR Techniques

There was no correlation between three techniques. Lateralization abilities of these MR modalities were evaluated by comparing the findings with electroclinical data. Threshold values derived from ±2 SD of the mean right/left ratios of control subjects. In spectroscopic values NAA/(Cho+Cr) ratios were used for lateralization. Lateralization was acquired in 4 of 7 patients (57%) in right TLE group and 1 of 7 patients (14%) in left TLE group. DTI studies lateralized 57% of patients in right TLE group and 28% of patients in left TLE group by right/left ratio of FA values. According to right/left ratio, in right TLE group 4 of 7 patients (57%) and in left TLE group 6 of 7 patients (85%) were lateralized.

Combining NAA/(Cho+Cr) with volumetric analysis increased lateralization to 71% in right TLE group and 85% in left TLE group. Likewise NAA/(Cho+Cr) and DTI together lateralized patients of 85% in right TLE group and 42% in the left-sided one. Finally the highest lateralization ratios such as 85% in right TLE group and 100% in left TLE group were obtained with the combination between DTI and volumetric analysis (Table 2). Utilizing all modalities together made no contribution to the accuracy of the DTI and volumetric analyses combination. The sensitivity and specificity ratios of all techniques for each hippocampus are summarized in Table 3.

### 3.5. Relations with Clinical Data

The clinical data (age, duration of epilepsy, febrile convulsion history, and response to AEDs) were compared with qMR findings. The quantitative data showed no association either with age or with response to AEDs. However a significant negative relationship (*p* = 0.016, *r* = −0.847) was observed between ipsilateral FA values and duration of epilepsy. Febrile convulsion history of patients showed significant positive correlation (*p* = 0.015, *r* = 0.851) with ipsilateral ADC values and negative correlation (*p* = 0.047, *r* = −761) with ipsilateral NAA/(Cho+Cr) ratios.

## 4. Discussion 

In this study, we investigated the relative lateralizing ability of single-voxel ^1^H MRS, volumetry, and DTI in a homogenous group of patients with lateralized EEG findings, qMR, and their clinical correlation in TLE patients. The comparison of qualitative and quantitative techniques was made on hippocampal atrophy. The qualitative analyses of visual inspection revealed insufficient results compared to objective volumetric analysis on 3D images that discloses variability of visual analyses which depends on subjective observations. The volumetric technique showed highest sensitivity and specificity ratios between all of the techniques (Table 3). The individualized left-right measurement difference may contribute to the increased sensitivity of lateralization in this study. Decreased prominent hippocampal volumes with highest lateralization ratios suggest both reason and results of epilepsy causing tissue loss.

Neuronal cell loss is an essential feature of mesial temporal sclerosis. Hippocampal NAA signals and NAA/(Cho+Cr) ratios were lower ipsilateral to epileptogenic side in compatible with other studies of ^1^H MRS in TLE [15, 16, 29]. The superiority of NAA reduction over Cho and Cr changes indicates predominant neuronal involvement, either by cellular loss or by metabolic impairment. In this study Cho values of ipsilateral hippocampus were increased significantly in right TLE group that was concordant with previous investigations [29]. It may be due to high Cho elevation in the early gliotic process of the hippocampus before evident signal intensity changes. NAA/(Cho+Cr) showed no relationship with volumetry, concordant with previous comparative reports [14, 15, 17]. MRS should be performed with chemical shift imaging (CSI) multivoxel spectroscopy. In both single-voxel spectroscopy (SVS) and CSI studies on TLE the lateralization ratios were reported to be similar to NAA/(Cho+Cr) ratios [17, 30–32]. Hsu et al. compared SVS with CSI in patients with complex partial seizures and also reported similar NAA/(Cho+Cr) ratios [33].

DTI characteristics of hippocampus showed significant reduction of FA in right TLE patient group compared to normal subjects concordant with previous data [9]. Some authors speculated that these diffusion abnormalities beyond the clearly identified epileptogenic zone are likely to correlate with structural changes such as neuronal loss and gliosis [34]. Another potential explanation is functional alteration as related to ongoing seizures. A deficit of myelin may explain the lower FA and additionally Cho impairment of ipsilateral epileptogenic hippocampus [29]. In the present study data did not concur exactly with some of published works which indicates ADC increment more significant in TLE patients [10, 11]. However in 4 of our 14 patients (28%) ADC was increased compared to the control group. The hippocampal FA decrement gives rise to the thought that epilepsy causes an impairment of tissue connectivity. Increased ADC in hippocampal formations might reflect that the cellular integrity of the tissue is not significantly affected, but diffusion is increased subjectively as is the function deficit caused by this situation. This disparity across studies reflects great variations in the methodology and in the number of cases. We believe that the selection of ROI on three consecutive thin (1 mm) slices from hippocampal body is likely to improve diagnostic accuracy and avoid partial-volume effects. The reproducible results with applying the same method several times on the same subject reflect reliability of our manual method.

Although our study consisted of unilateral TLE patients according to EEG findings, bilateral hippocampal sclerosis may be present in several patients. In the present study contralateral MRS, volumetric and diffusion abnormalities may reflect the micro- and macrostructural changes of early hippocampal involvement which is not prominent on conventional MRI and EEG findings.

To our knowledge, this is the first study that evaluated DTI values, ^1^H MRS findings, and volumetric measurements comparatively in a selected TLE patients group. The previous diffusion studies found no association between increased diffusivity and the degree of hippocampal atrophy [30, 31]. Likewise in these findings, hippocampal DTI characteristics showed no correlation with either volumetry or NAA/(Cho+Cr) in our study. It seems that all the diffusion abnormalities may not be explained exactly by neuronal loss and metabolic changes in TLE. The distinct outcomes from hippocampal tissue could be the possible explanation of the difference between three methods. Volumetry reflects the macrostructural alterations such as tissue atrophy, while MRS gives information about metabolic conditions and DTI investigates tissue integrity in microstructures. So the measurements obtained from the different techniques can be considered as significantly asymmetric.

On the other hand performing these quantitative methods together improved the diagnostic accuracy of lateralizing in TLE patients. In particular combining DTI with volumetry provided the best lateralizing ratios (85%–100%), while MRS had relatively lower support (57%–14%). In clinical management these data's combination may indicate the involvement of hippocampus in preoperative process of TLE patients.

Clinical aspects of this study represented that age of onset of epilepsy and response to AEDs have not clear effects on ipsilateral hippocampus. Some investigators found a significant relationship between hippocampal volumetry and a history of febrile convulsion in early childhood before [12, 35]. Unlike this, in our data the history of febrile convulsions associated significantly with ipsilateral increased ADC values and decreased NAA/(Cho+Cr). An increase in extracellular space and NAA reduction may be possible results of hippocampal damage related to prolonged febrile convulsions. Duration of epilepsy correlated significantly only with ipsilateral FA values. FA values decreased proportionally with longer duration of epilepsy. Structural damage through neuronal loss and gliosis may be possible explanations of this finding.

The present study has several limitations. First, all patients were treated with multiple antiepileptic drugs for a long time. A published research speculated the transient increased diffusivity in corpus callosum after taking antiepileptic therapy [36]. Therefore the chronic effects of these drugs on diffusion characteristics remain unknown. Secondly, the variation of duration of epilepsy in a wide range could affect the lateralization ratios with different stages of neuronal loss. Finally the study's gold standard is EEG and we do not know the pathologic data of the substrate that is being imaged. We observed lateralizing ability of these methods in a selected EEG lateralized group of patients with HS.

In conclusion the results of this study demonstrate that volumetry has higher diagnostic accuracy between qMR modalities in lateralizing TLE patients and should be performed in all patients for preoperative analysis. MRS with NAA/Cho+Cr ratio and DTI with FA values provide limited information to depict epileptogenic focus. The lack of correlation between these modalities reflects that they investigate analogous but distinct process in the investigated epileptogenic areas. However performing these techniques in combination, particularly DTI with volumetry, improves the diagnostic accuracy of lateralization. These additional qMR modalities may be helpful on patients particularly who cannot be lateralized by conventional MR images and EEG findings. Further comparative studies are needed to reveal the role of quantitative MR modalities in patients with pathological and negative MR findings.

## Figures and Tables

**Table 1:** Summary of patient population.

Patient	EEG	R HF signal	L HF signal	Duration	Age	AEDs	Response to AEDs	History of FC
1	R TLE	Increased	Normal	23 years	24	CBZ+TPX	(−)	(+)
2	R TLE	Increased	Normal	39 years	40	CBZ+LEV+VPA	(+)	(+)
3	R TLE	Increased	Increased	23 years	23	VPA	(−)	(−)
4	R TLE	Increased	Normal	1 year	31			(+)
5	R TLE	Increased	Increased	7 years	37	CBZ	(+)	(+)
6	R TLE	Increased	Increased	28 months	66	CBZ	(+)	(−)
7	R TLE	Increased	Normal	22 years	35	CBZ+LEV	(−)	(+)
8	L TLE	Increased	Increased	8 years	26	CBZ	(+)	(+)
9	L TLE	Normal	Increased	15 years	36	CBZ+VPA	(−)	(+)
10	L TLE	Normal	Normal	6 years	61	CBZ	(+)	(−)
11	L TLE	Normal	Increased	18 years	19	PHE+VPA	(−)	(+)
12	L TLE	Increased	Increased	8 years	29	VPA	(−)	(−)
13	L TLE	Normal	Increased	10 years	25	VPA	(+)	(−)
14	L TLE	Normal	Normal	6 years	27	CBZ	(−)	(−)

**Table 2:** Lateralizing ability of quantitative MR modalities.

	Right TLE	Left TLE	All patients
Volumetry	57%	85%	71%
Spectroscopy	57%	14%	35%
DTI	57%	28%	42%
Volumetry +	71%	85%	78%
spectroscopy			
Spectroscopy +	85%	42%	64%
DTI			
DTI + volumetry	85%	100%	92%

**Table 3:** The sensitivity and specificity ratios of all techniques for each hippocampus.

		Right TLE	Left TLE
MRS	Sensitivity (%)	57	14
	Specificity (%)	100	100
DTI	Sensitivity (%)	57	28
	Specificity (%)	100	100
Volumetry	Sensitivity (%)	57	85
	Specificity (%)	100	100
